# Targeting CD73 limits tumor progression and enhances anti-tumor activity of anti-PD-1 therapy in intrahepatic cholangiocarcinoma

**DOI:** 10.1007/s00432-024-05869-1

**Published:** 2024-07-13

**Authors:** Bao-Ye Sun, Dai Zhang, Wei Gan, Jing-Fang Wu, Zhu-Tao Wang, Guo-Qiang Sun, Jian Zhou, Jia Fan, Yong Yi, Bo Hu, Bo-Heng Zhang, Shuang-Jian Qiu

**Affiliations:** 1 Department of Liver Surgery and Transplantation, Zhongshan Hospital, Fudan University, Shanghai, 200032 China; 2 Department of Hepatic Oncology, Xiamen Clinical Research Center for Cancer Therapy, Zhongshan Hospital, Fudan University (Xiamen Branch), Xiamen, 361015 China; 3 Department of Hepatic Oncology, Liver Cancer Institute, Key Laboratory for Carcinogenesis and Cancer Invasion (Ministry of Education), Zhongshan Hospital, Fudan University, Shanghai, 200032 China; 4 Department of Pancreatic Surgery, Zhongshan Hospital, Fudan University, Shanghai, 200032 China

**Keywords:** Intrahepatic cholangiocarcinoma, CD73, Immunotherapy, Combination therapy

## Abstract

**Background & aims:**

Patients with intrahepatic cholangiocarcinoma (iCCA) respond poorly to immune checkpoint blockades (ICBs). In this study, we aimed to dissect the potential mechanisms underlying poor response to ICBs and explore a rational ICB-based combination therapy in iCCA.

**Methods:**

scRNA-seq dataset GSE151530 was analyzed to investigate the differentially expressed genes in malignant cells following ICBs therapy. RNA-seq analysis and western blot assays were performed to examine the upstream and downstream signaling pathways of CD73. Subcutaneous tumor xenograft models were utilized to investigate the impact of CD73 on iCCA growth. Plasmid AKT/NICD-induced spontaneous murine iCCAs were used to explore the therapeutic efficacy of CD73 enzymatic inhibitor AB680 combined with PD-1 blockade. Time-of-flight mass cytometry (CyTOF) was conducted to identify the tumor-infiltrating immune cell populations and their functional changes in murine iCCAs treated with AB680 in combination with PD-1 antibody.

**Results:**

scRNA-seq analysis identified elevated CD73 expression in malignant cells in response to ICBs therapy. Mechanistically, ICBs therapy upregulated CD73 expression in malignant cells via TNF-α/NF-κB signaling pathway. In vivo studies revealed that CD73 inhibition suppressed the growth of subcutaneous tumors, and achieved synergistic depression effects with gemcitabine and cisplatin (GC). Adenosine produced by CD73 activates AKT/GSK3β/β-catenin signaling axis in iCCA cells. CD73 inhibitor AB680 potentiates anti-tumor efficacy of PD-1 antibody in murine iCCAs. CyTOF analysis showed that AB680 combined with anti-PD-1 therapy promoted the infiltration of CD8^+^ T, CD4^+^ T cells, and NK cells in murine iCCAs, while simultaneously decreased the proportions of macrophages and neutrophils. Moreover, AB680 combined with anti-PD-1 significantly upregulated the expression of Granzyme B, Tbet and co-stimulatory molecule ICOS in infiltrating CD8^+^ T cells.

**Conclusions:**

CD73 inhibitor AB680 limits tumor progression and potentiates therapeutic efficacy of GC chemotherapy or anti-PD-1 treatment in iCCA. AB680 combined with anti-PD-1 therapy effectively elicits anti-tumor immune response.

**Supplementary Information:**

The online version contains supplementary material available at 10.1007/s00432-024-05869-1.

## Introduction

Intrahepatic cholangiocarcinoma (iCCA) is a highly lethal primary liver malignancy and lacks effective treatment options, with increasing morbidity and mortality worldwide (PEERY A F et al. 2019; RIZVI and GORES 2017; KENNEDY L B and SALAMA A K S 2020). Most patients are diagnosed at advanced stages, missing the chance for surgery. Even for those undergoing curative resection for iCCA, the 5-year survival rate remains only 14-40% (ENDO et al. 2008; PEERY A F et al. 2021). In recent years, Gem/Cis (gemcitabine/cisplatin) chemotherapy combined with anti-PD-L1/PD-1 therapy has been recommended as the first-line regimen for advanced iCCA (MOK T S K, WU Y L, KUDABA et al. 2019; UENO et al. 2019). However, the overall response rate of this combination therapy remains suboptimal, with limited survival benefit. The high incidence of tumor relapse and metastasis impairs the therapeutic efficacy of surgery, chemotherapy, and immunotherapy. Therefore, it is urgent to identify novel targets that could enhance the therapeutic efficacy of current treatment regimens for iCCA.

Over the past decade, immune checkpoint blockades (ICBs) particularly through targeting the immune-inhibitory PD-L1/PD-1 pathway have shown promising anti-cancer activity in several solid malignancies. However, the majority of iCCA patients fail to respond to anti-PD immunotherapy due to innate resistance, adaptive resistance or progressive disease after ICBs treatment (SALKENI M A, SHIN J Y, GULLEY 2021). Clinical evidence has shown that compared with standard Gem/Cis chemotherapies, anti-PD-L1/PD-1 therapy could hardly induce a higher objective response or bring survival benefit to patients with advanced iCCA (YANG et al. 2022; SPIOTTO M T, ROWLEY D A, SCHREIBER 2004). A thorough investigation of mechanisms underlying tumor resistance to anti-PD therapy would be valuable in exploring more effective anti-cancer combination therapies for iCCA.

We have previously dissected the intra-tumoral changes following ICBs therapy in iCCA ecosystem via single-cell analysis, including the enhanced VEGF signaling between fibroblasts and endothelial cells, upregulation of pro-M2 polarization genes such as SPP1, APOE, CTSB, and MARCO in TAMs, and upregulated CD73 expression in malignant cells (SUN B Y, ZHOU et al. 2022; SUN B Y et al. 2023). These findings revealed the potential reasons for the poor efficacy of ICBs monotherapy in iCCA. Among these therapeutic targets, CD73, also known as ecto-5’-nucleotidase (NT5E), can catalyze AMP into adenosine. The adenosine generated by CD73 functions as a potent immunosuppressor in tumor ecosystem and plays a crucial role in tumor immune evasion (ANTONIOLI et al. 2013; PANG et al. 2021). Targeting extracellular adenosine signaling pathway have shown promising anti-tumor effect in several preclinical models (ALLARD B et al. 2017; LI et al. 2018). Notably, selective enzymatic inhibitors or blocking monoclonal antibodies against CD73 have been devised and been evaluated under clinical trials (VIGANO et al. 2019; ALLARD and ALLARD 2020). Moreover, our previous study revealed that CD73 promotes malignant behaviors of iCCA cells and CD73 high expression is associated with poor prognosis and an immunosuppressive tumor microenvironment in iCCA. Therefore, it would be of great value to further explore the therapeutic effects of CD73 inhibition on iCCA initiation and progression.

In this study, we sought to investigate whether targeting CD73 could potentiate the therapeutic efficacy of anti-PD-1 therapy or Gem/Cis chemotherapy using both immunocompetent or immunodeficient mouse iCCA models. We found that ICBs therapy could upregulate CD73 expression in malignant cells via TNF-α/NF-κB signaling and adenosine produced by CD73 activated AKT/GSK3β/β-catenin signaling axis in iCCA cells. CD73 inhibition suppressed the growth of subcutaneous tumors in nude mice, and achieved synergistic suppressive effects with GC chemotherapy. CD73 inhibitor AB680 potentiates the efficacy of anti-PD-1 antibody in murine spontaneous iCCAs and this combination therapy efficiently elicits anti-tumor immune response as revealed by CyTOF analysis. Our results identified CD73 as a promising therapeutic target for iCCA.

## Materials and methods

### Patient cohorts selected

This study included five iCCA patient cohorts. (1) We analyzed the scRNA-seq data of 12 iCCA samples receiving ICBs therapy from GSE151530 and divided them into two groups (Pre-treatment and On-treatment group) (MA et al. 2021). (2) Another single-cell dataset GSE125449 (MA et al. 2019) was used to investigate the expression level of CD73 on various cell types in iCCA. (3) The FU-iCCA cohort enrolled 244 iCCA patients with complete follow-up information from Zhongshan Hospital, Fudan University (DONG et al. 2022). RNA sequencing data of surgically resected iCCA samples from this cohort were analyzed. (4) ICGC cohort (JUSAKUL et al. 2017): The GSE89749 dataset was downloaded from the Gene Expression Omnibus (GEO) database. (5) The CCA protein cohort included proteome profiling data of 210 primary formalin-fixed, paraffin-embedded (FFPE) CCA samples (DENG and RAN 2023).

### Single-cell data processing

scRNA-seq datasets GSE151530 (MA et al. 2021)and GSE125449 (MA et al. 2019) were analyzed via R package Seurat v4 (SATIJA et al. 2015) as previously described (SUN B Y, ZHOU et al. 2022). After normalization and principal component analysis (PCA) of the 2000 highly variable genes, the top 20 PCs were selected for the clustering of all cells. The cell types were manually annotated according to the highly expressed genes.

### Cell lines

Human iCCA cell lines RBE, HCCC-9810, and CCLP1 were obtained from the Liver Cancer Institute, Fudan University. RBE and HCCC-9810 cells were cultured in RPMI 1640 medium containing 10% FBS in a 37 °C humidified incubator with 5% CO_2_. CCLP1 cells were cultured in high glucose DMEM supplemented with 10% FBS.

### RT-qPCR

Total RNA was extracted using TRIzol (Invitrogen) method. Reverse transcription (RT) and qPCR were performed using Prime Script RT Master Mix and TB Green PCR kit (Takara, Japan). β-actin was set as the internal control. The 2^−ΔΔCt^ method was applied to quantify the relative mRNA expression levels of indicated genes (CUI et al. 2022). The following primers were used: CD73, 5′-TTAGGACCTGGCTTTGTG-3′ (F), 5′-GTTGCTGACCCTGAGTAATC-3′ (R); β-actin, 5′-CACCATTGGCAATGAGCGGTTC-3′ (F), 5′-AGGTCTTTGCGGATGTCCACGT-3′ (R).

### Western blot

Western Blot was performed as described before (SUN B Y et al. 2023). Briefly, cells were lysed in RIPA lysis buffer added with protease and phosphatase inhibitors (Beyotime, China). Protein samples were separated by 10% SDS-PAGE gel, electro-transferred onto PVDF membranes, and then incubated with primary antibodies and HRP-conjugated secondary antibody. β-actin was used as a loading control (JIN-YI Z et al. 2023). The following antibodies were used: anti-CD73 (1:1000, Cat# 13,160 S, CST), anti-AKT (1:1000, Cat# ab8805, Abcam), anti-p-AKT (1:1000, Cat# ab38449, Abcam), anti-GSK3β (1:1000, Cat# 12456 S, CST), anti-p-GSK3β (1:1000, Cat# 9323, CST), anti-β-catenin (1:1000, Cat# 8480 S, CST), anti-p65 (1:1000, Cat# 8242, CST), anti-p-p65 (1:1000, Cat# 3033 S, CST), anti-IκBα (1:1000, Cat# 4812 S, CST), anti-p-IκBα (1:1000, Cat# 9246 S, CST), and β-actin (1:1000, Cat# 4970 S, CST).

### Lentivirus-mediated construction of stable cell lines

For CD73 overexpression, the coding sequence of human CD73 gene was cloned into the lentiviral vector pLVX-CD73 by Genechem (Shanghai, China). For CD73 knockdown, shCD73 Oligos were cloned into the pLVX-Puro vector (GeneChem, Shanghai). Cells transfected with lentiviruses underwent selection with 5µg/ml of puromycin. shCD73 Oligos sequences are as following: shCD73: 5’-AGCAGCATTCCTGAAGATC-3’;

### RNA-seq

The TRIzol method was used to extract total RNA of RBE empty vector and CD73-KD cells according to the manufacturer’s protocol. RNA-Seq (*n* = 3) was performed on an Illumina Hiseq 2000 platform. The transcriptome sequencing and analysis were conducted by OE biotech, Shanghai. The raw RNA-seq transcripts were normalized in fragments per kilobase of transcript per million mapped reads (FPKM) and used for pathway enrichment analysis.

### Animal care and use

Animal studies were conducted according to the protocols approved by the Institutional Animal Care and Use Committee of Zhongshan Hospital, Fudan University. Six-week-old nude mice and C57BL/6 mice were purchased from Shanghai Ji-hui. All mice were housed under specific pathogen–free conditions in the animal center of Fudan University.

### AKT/NICD-induced murine iCCA model

To establish an AKT/NICD-induced spontaneous iCCA model (YAMAMOTO et al. 2017; DIGGS L P, RUF 2021), 20 µg PT3-myr-AKT-HA (Addgene plasmid #31,789), 20 µg pT3-EF1a-NICD (Addgene plasmid #46,047), and 10 µg Sleeping Beauty SB100x transposase-encoding plasmid (Addgene plasmid #34,879) per mouse dissolved in 2 mL Ringer solution were injected into C57BL/6 female mice through tail vein within 5 s.

### Subcutaneous xenograft tumor model

1 × 10^7^ CCLP1 cells suspended in 200µL PBS per mouse were injected subcutaneously into the left flank of six-week-old male nude mice (ZHAO et al. 2020). Two weeks after tumor cell implantation, mice were randomized and received indicated treatment. Tumor volume was measured by caliper every 3–4 days and calculated using the formula length (mm) × width^2^ (mm)/2. At the experimental endpoint, mice were sacrificed and tumors were harvested for further analyses.

### Treatments

For CD73 enzymatic activity inhibitor AB680 (MedChemExpress) administration, the stocks were dissolved in 100% DMSO. AB680 was diluted in 10%DMSO + 90% SBE beta cyclodextrin (SBE-b-CD) in 0.9% NaCl. Two weeks after tumor induction, AB680 (10 mg/kg) was administered daily via the intraperitoneal route until the end of the experiment. For anti-PD-1 therapy, mice were injected intraperitoneally with 10 mg/kg anti-PD-1 monoclonal antibody (BioXCell, BE0146) or isotype control monoclonal antibody (BioXCell, BE0089) every 3 days following the treatment schedule shown in Fig. 5A. For Gem/Cis chemotherapy, mice were treated with weekly intraperitoneal (i.p) injection of gemcitabine (MedChemExpress, 100 mg/kg) and cisplatin (MedChemExpress, 4 mg/kg) dissolved in 100µL PBS as described before (DIGGS L P, RUF 2021). Mice were routinely monitored to examine the potential adverse effects of tumor growth and treatment. At the endpoint of the indicated therapy, mice were sacrificed and tumor tissues were harvested for further analyses.

### Immunohistochemistry (IHC) staining

IHC staining of the tissue slides was performed according to the procedures detailed before (GAO Q, QIU S J, FAN et al. 2007). After blocking endogenous peroxidase and antigen retrieval, the slides were incubated with primary antibodies CK19 (1:1000, Cat# ab52625, Abcam) and CD73 (1:500, Cat# 13,160 S, CST).

### CyTOF data analysis

Time-of-flight mass cytometry (CyTOF) was performed by PLTTech Inc. (Hangzhou, China) according to a previously described protocol (HU et al. 2022). The 42 metal-labeled antibody panel was devised to assess the expression of cell lineage markers and function-related markers on CD45^+^ cells from dissociated tumors. Briefly, single-cell suspensions of freshly harvested tumors were obtained using Mouse Tumor Dissociation Kit (Miltenyi Biotec; #130-096-730). Samples were stained with metal-labeled antibodies and signal detection was performed on a Helios instrument (Fluidigm). Antibody clones are listed in Supplementary Table S1. The gating strategy was set as live and single immune cells (CD45^+^). The tSNE plots and heatmaps were applied to characterize immune cell clusters after normalization and nonlinear dimensionality reduction of original data.

### Differential gene expression analysis

For single-cell data analysis, differentially expressed genes (DEGs) of single malignant cells before and after ICBs therapy was conducted using the ‘‘FindMarkers’’ function in Seurat package, with log_2_(fold change) ≥ 0.585 and P value < 0.01.

### Gene set enrichment analysis

Gene Set Enrichment Analysis (GSEA) (SUBRAMANIAN et al. 2005) was performed to analyze the differential Hallmark gene sets between control and shCD73 RBE cells.

### Statistical analysis

Statistical analyses were performed using R version 4.1.2 and Graphpad 9.0 software. Student’s *t* test or Mann–Whitney test was used to compare the differences of continuous variables between two groups. If variances within two groups were not homogeneous, a nonparametric Wilcoxon rank-sum test was used. Prognostic value of CD73 was assessed by Kaplan-Meier overall survival (OS) analyses and log-rank tests using R packages survival and survminer. P value less than 0.05 was considered as statistically significant.

## Results

### CD73 expression in malignant cells was significantly upregulated in response to ICBs therapy

**Fig. 1:**
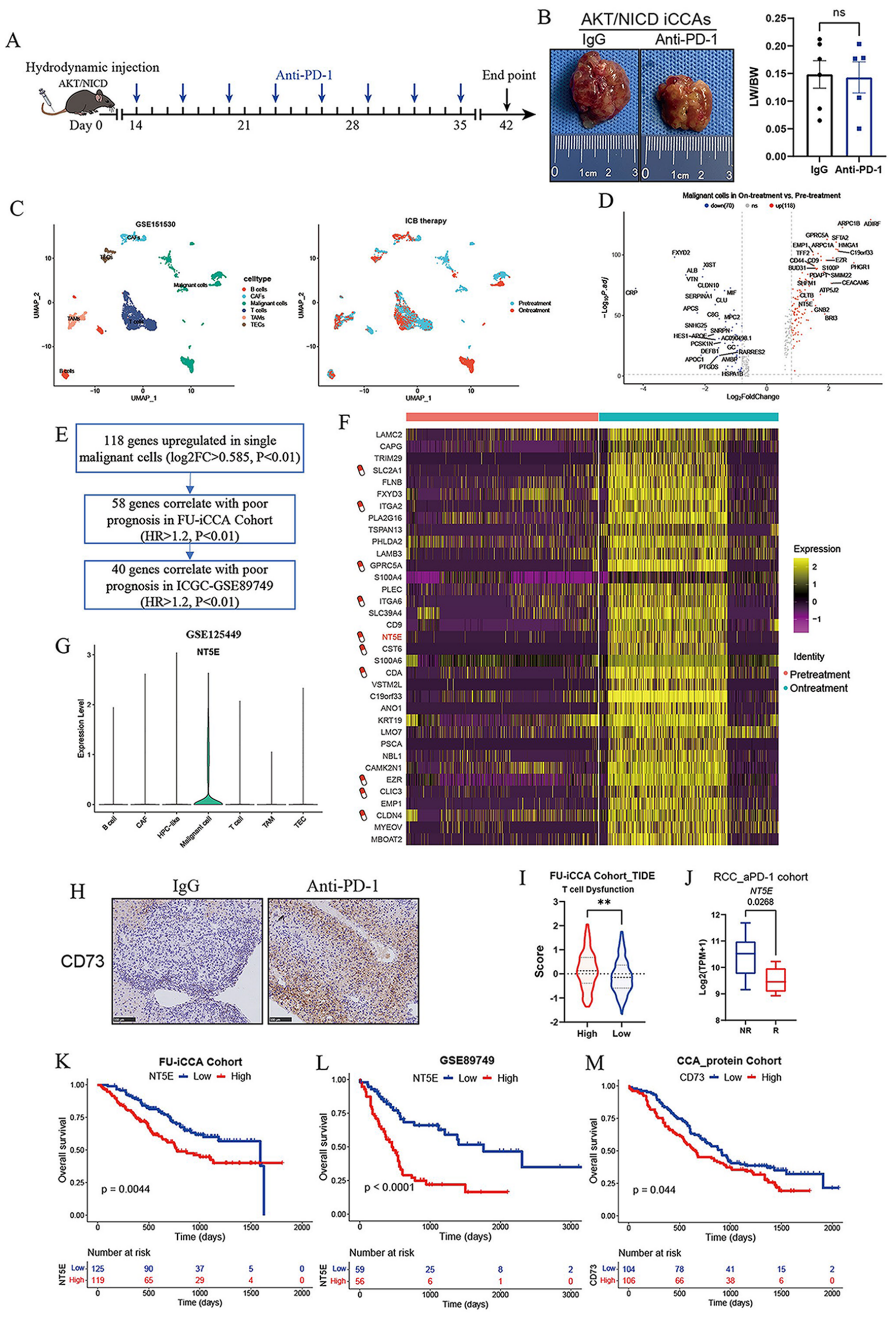
Elevated CD73 expression of malignant cells in response to ICBs immunotherapy. (**A**) Schematic representation of the treatment schedule for anti-PD-1 monotherapy in AKT/NICD-induced spontaneous murine iCCAs. (**B**) Representative gross images and statistical results from AKT/NICD-induced murine iCCAs that received indicated treatments (6 mice for IgG and 5mice for anti-PD-1 treatment, 10 mg/kg). (**C**) scRNA-seq analysis of GSE151530 showing 12 iCCA biopsies from 10 patients collected at baseline or during ICBs therapy (PD-1 or PD-L1/CTLA-4). UMAP plot showing single cells distinguished by cell types and cell origins from different biopsy time points. (**D**) Volcano plot showing differentially expressed genes in malignant cells before and after ICBs treatment. (**E**) 40 genes that were upregulated in single malignant cells following ICBs therapy were associated with poor prognosis in both FU-iCCA Cohort and ICGC Cohort. (**F**) The drug-gene interaction database (DGIdb) database was used to identify the potential drug targets among the indicated genes. (**G**) Violin plot showing the expression levels of CD73 in different cell types in iCCA from GSE125449. (**H**) Immunohistochemical staining images showing CD73 expression in AKT/NICD iCCAs that received IgG or anti-PD-1 treatment. (**I**) Analysis of T cell dysfunction signature scores by TIDE algorithm in patients with high or low CD73 expression in FU-iCCA cohort. (**J**) Expression levels of CD73 between Non-Responsive versus Responsive (NR vs. R) samples to anti-PD-1 monotherapy in RCC cohort. Kaplan-Meier survival curves of OS for patients grouped by CD73 mRNA expression level in FU-iCCA cohort (**K**) and ICGC cohort (**L**), as well as CD73 protein levels in CCA protein cohort (**M**). **P < 0.01; OS, overall survival; iCCA, intrahepatic cholangiocarcinoma

Clinical trials of anti-PD therapy in advanced CCA have so far failed to show a higher treatment response compared with standard chemotherapies. We first tested the therapeutic efficacy of anti-PD-1 therapy in immunocompetent murine iCCAs. Hydrodynamic tail vein injection was performed to deliver plasmids encoding for AKT and NICD (activated form of NOTCH) into C57BL/6 mouse livers, a well-established murine spontaneous iCCA model (FAN et al. 2012). Compared to control IgG, treatment with anti-PD-1 therapy had limited effect on tumor formation and progression (Fig. 1A-B), indicating that murine iCCAs have poor response to anti-PD-1 monotherapy. To explore the potential mechanisms underlying poor response to ICBs and explore a more rational ICB-based therapy in iCCA, we analyzed the scRNA-seq dataset GSE151530 (MA et al. 2021), which included 12 iCCA biopsies collected at different time points during ICBs therapy. We annotated six major cell subsets using known marker genes, including B cells, CAFs, Malignant cells, T cells, TAMs, and TECs (Fig. 1C). Next, we performed differential analysis of malignant cells from ICBs-treated group and untreated group, and found that 118 genes were upregulated in single malignant cells following ICBs therapy (Fig. 1D). Among them, 40 genes were associated with poor prognosis in both FU-iCCA Cohort and ICGC Cohort (Fig. 1E). The drug-gene interaction database (DGIdb) database was then used to identify the potential drug targets in the screened genes (Fig. 1F), and NT5E/CD73 was reported to be associated with poor prognosis in a variety of tumors. CD73 encoded by NT5E gene can catalyze AMP to adenosine, and adenosine produced by hydrolysis can induce immunosuppression and angiogenesis, which is a novel target for tumor immunotherapy. Single cell atlas showed high expression of CD73 on malignant cells (Fig. 1G). Immunohistochemical analysis showed that CD73 expression was up-regulated in iCCA tissues of mice treated with PD-1 antibodies (Fig. 1H). High expression of CD73 is associated with T cell dysfunction in FU-iCCA Cohort and anti-PD-1 treatment resistance in the renal cell carcinoma (RCC) cohort (Fig. 1I-J). Kaplan-Meier survival analyses showed that in FU-iCCA cohort and ICGC cohort, patients with high CD73 mRNA expression had significantly shorter OS (Fig. 1K-L). In CCA protein cohort (DENG and RAN 2023), patients with higher CD73 protein level based on the proteomic data had worse prognosis (Fig. 1M). Taken together, these data suggested that upregulated CD73 expression in malignant cells following ICBs therapy could account for the poor response to ICBs in iCCA.

### ICBs therapy upregulates CD73 expression in tumor cells through TNF-α/NF-κB signaling pathway

**Fig. 2:**
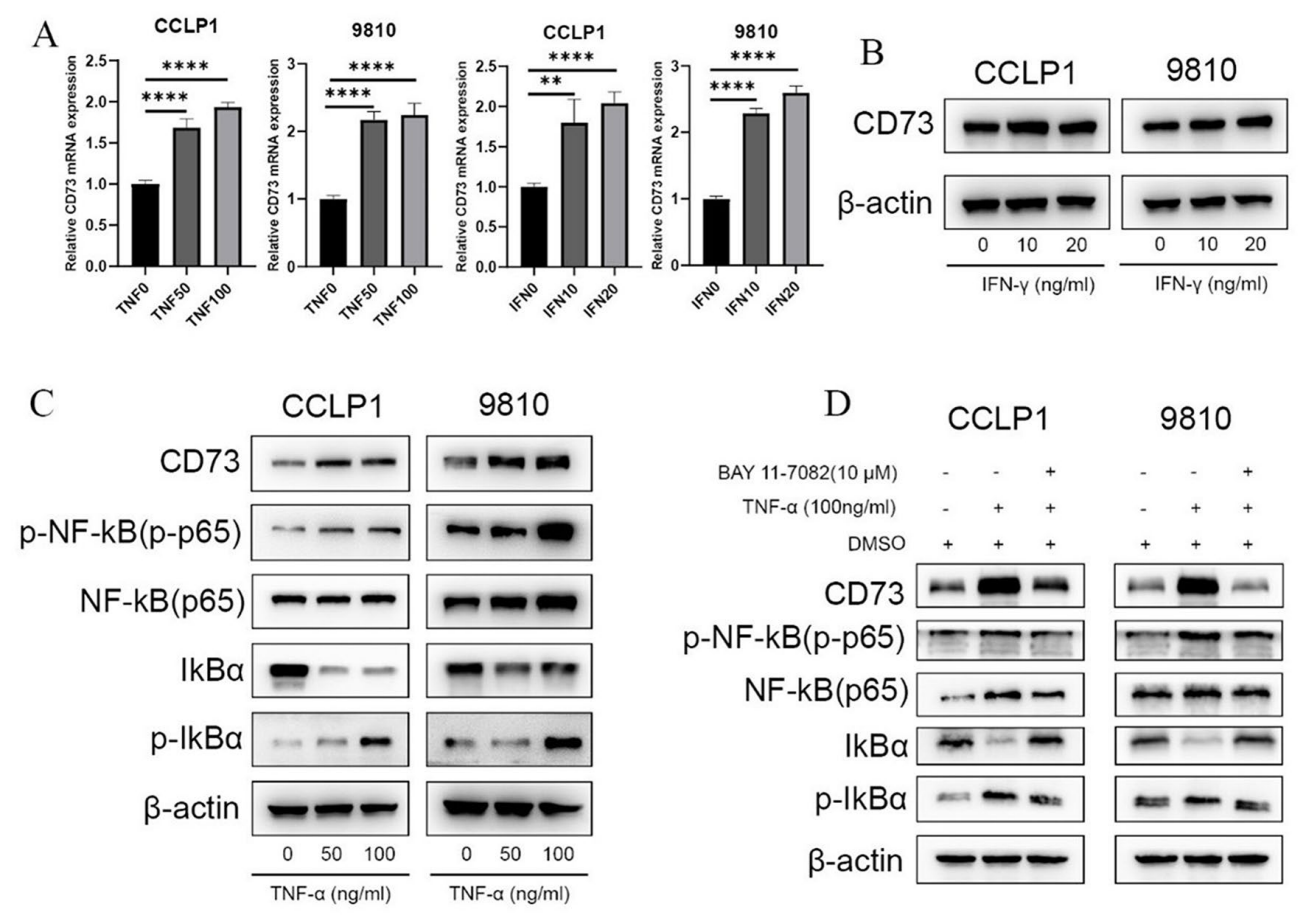
ICBs therapy upregulates CD73 expression in iCCA cells through TNF-α/NF-κB signaling pathway. (**A**) Evaluation of the effect of TNF-α and IFN-γ on CD73 mRNA expression in human iCCA cells CCLP1 and 9810 as determined by RT-PCR assays. (**B**) Evaluation of the effect of IFN-γ on CD73 protein expression in iCCA cells as detected by WB assays. (**C**) WB assays evaluating the effect of different concentrations of TNF-α on CD73 protein expression, as well as the downstream NF-κB signaling pathway in iCCA cells. (**D**) WB assays evaluating the effect of TNF-α on CD73 expression and NF-κB signaling in iCCA cells, with or without NF-κB pathway inhibitor BAY11-7082. WB, western blot; *P < 0.05, **P < 0.01, ***P < 0.001

Next, we explored the underlying mechanism by which CD73 expression is upregulated in malignant cells after ICBs treatment. Given that ICBs immunotherapy promotes the secretion of IFN-γ, TNF-α and other inflammatory cytokines in tumor microenvironment (WU et al. 2022; ZHANG et al. 2020), we first assessed the influence of cytokines IFN-γ and TNF-α on the expression of CD73 in tumor cells. Treatment with TNF-α and IFN-γ increased the expression of CD73 mRNA in iCCA cells (Fig. 2A), whereas no significant changes in protein levels of CD73 were detected after IFN-γ stimulation (Fig. 2B). Meanwhile, addition of TNF-α promoted CD73 protein expression and activated the downstream NF-κB signaling pathway in iCCA cells (Fig. 2C). Moreover, TNF-α-induced CD73 upregulation was abrogated by the treatment with NF-κB pathway inhibitor BAY11-7082 (Fig. 2D), indicating that ICBs therapy could upregulate CD73 expression in iCCA cells through TNF-α/NF-κB signaling pathway.

### CD73 inhibitor AB680 combined with GC chemotherapy exerts a synergistic anti-tumor effect

**Fig. 3:**
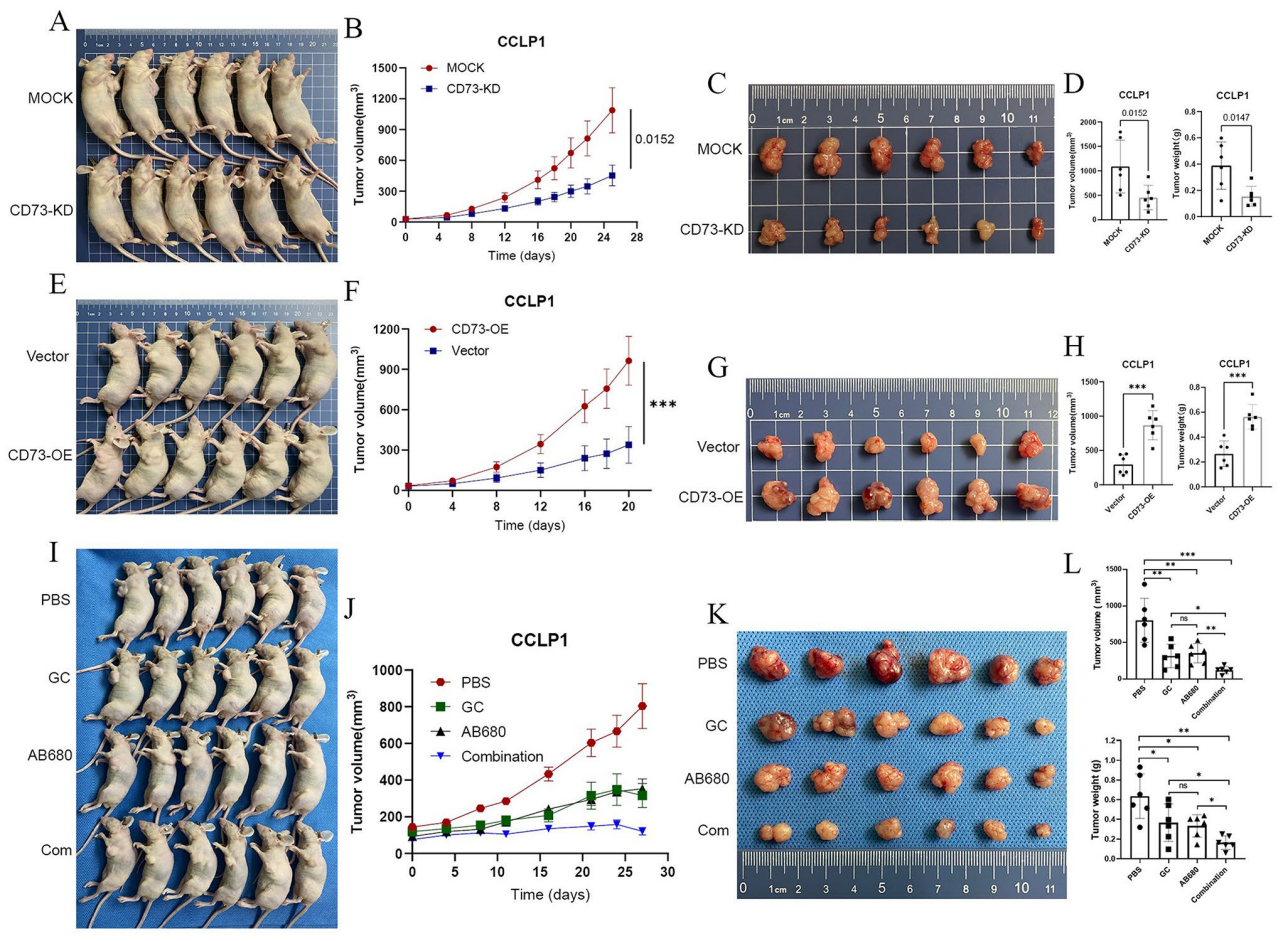
CD73 inhibitor AB680 synergizes with GC chemotherapy to inhibit CCLP1 tumor growth in vivo. (**A**) Establishment of the subcutaneous xenograft model with nude mice using sh-MOCK or CD73-knockdown CCLP1 cells. Growth curves (**B**), gross images (**C**), volumes and weight (**D**) of CCLP1 subcutaneous tumors from indicated groups. (**E**) Subcutaneous xenograft model with nude mice using vector or CD73-OE CCLP1 cells. Growth curves (**F**), gross images (**G**), volumes and weight (**H**) of CCLP1 subcutaneous tumors from indicated groups. (**I**) Subcutaneous CCLP1 tumor-bearing nude mice receiving PBS, Gem/Cis chemotherapy, CD73 inhibitor AB680, or combination therapy. Growth curves (**J**), gross images (**K**), volumes and weight (**L**) of CCLP1 tumors from indicated treatment groups. *P < 0.05, **P < 0.01, ***P < 0.001

Our previous experiments showed that CD73 promoted iCCA cell proliferation, migration, invasion, and epithelial-mesenchymal transition in vitro (SUN B Y et al. 2023).To further investigate the effects of CD73 on iCCA growth in vivo, we established 3 subcutaneous xenograft tumor models. We found that CD73 knockdown markedly inhibited the growth of CCLP1 subcutaneous tumors (Fig. 3A-D), whereas CD73 overexpression promoted tumor growth (Fig. 3E-H). Chemotherapy with Gem/Cis (gemcitabine/cisplatin) remains the standard care for patients with CCA (VALLE et al. 2010). Either CD73 inhibitor AB680 or GC chemotherapy suppressed the growth of subcutaneous tumors. However, no significant difference in tumor growth inhibition was observed between the Gem/Cis- and AB680-treated mice (Fig. 3I-L). More importantly, combined treatment with AB680 and GC chemotherapy effectively showed a significantly stronger anti-tumor effect than either single agent alone (Fig. 3I-L), indicating that AB680 combined with GC chemotherapy had a synergistic anti-tumor effect. Addition of AB680 could enhance response to current GC chemotherapy in iCCA.

### CD73 activates the AKT/GSK3β/β-catenin signaling pathway in iCCA cells via adenosine

**Fig. 4:**
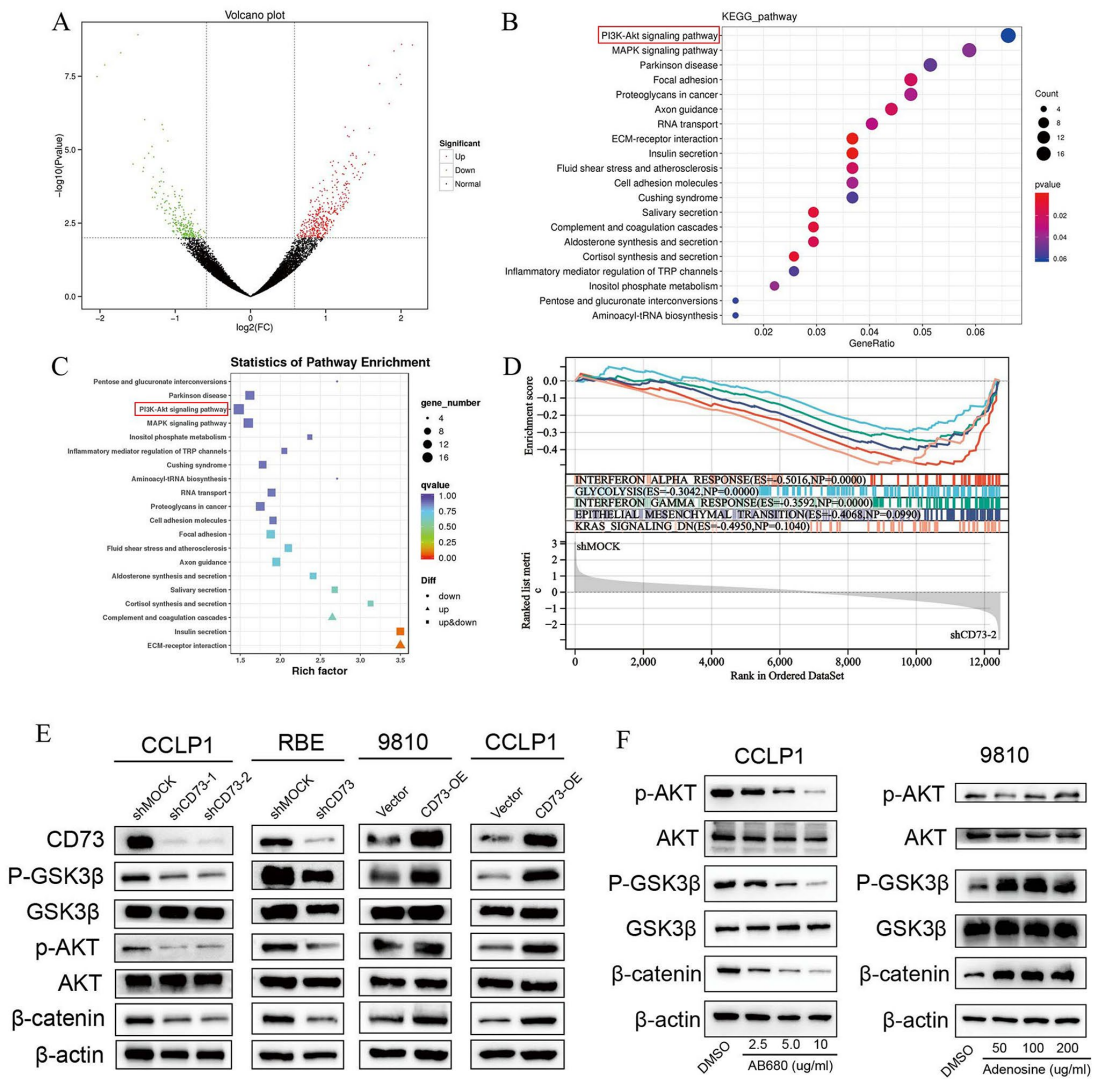
CD73 activates the AKT/GSK3β/β-catenin signaling pathway in iCCA cells via adenosine (**A**) Volcano plot showing differentially expressed genes in RBE cells transfected with shCD73 and shMOCK identified by RNA-seq analysis. (**B-C**) KEGG pathway analysis showing differential signaling pathways regulated by CD73 knockdown in RBE cells. (**D**) Gene set enrichment analysis (GSEA) showing that Hallmark gene sets Hypoxia, Inflammatory response, Glycolysis, Epithelial-mesenchymal transition pathways were enriched in shMOCK RBE cells compared with shCD73 cells. (**E**) Phosphorylation levels of AKT, GSK3β, and total β-catenin protein expression in the indicated iCCA cells as determined by WB assays. (**F**) Phosphorylation levels of AKT, GSK3β, and total β-catenin protein levels in CCLP1 cells treated with different concentrations of CD73 enzymatic activity inhibitor AB680 (left) or in 9810 cells under different concentrations of adenosine treatments (right) detected by WB assays

To analyze the potential mechanism underlying the oncogenic role of CD73 in iCCA, we silenced CD73 expression in human iCCA cells RBE using shCD73 and performed RNA-seq (Fig. 4A). Kyoto Encyclopedia of Genes and Genomes (KEGG) analysis demonstrated that the pathways regulated by shCD73 were involved in PI3K-AKT and MAPK signaling (Fig. 4B-C). GSEA analysis revealed that Hypoxia, Inflammatory response, Glycolysis, Epithelial-mesenchymal transition pathways were enriched in shMOCK RBE cells compared with shCD73 cells (Fig. 4D). Activation of PI3K-AKT signaling phosphorylates AKT and GSK3β, resulting in β-catenin accumulation, which then enters the nucleus and regulates genes related to cell division and growth. Western blot analysis showed that the protein levels of p-AKT, p-GSK3β and β-catenin decreased after CD73 knockdown in human iCCA cell lines, while CD73 overexpression in iCCA cell lines increased the protein levels of p-AKT, p-GSK3β and β-catenin (Fig. 4E). We next sought to investigate whether the enzymatic activity of CD73 was required for its function in activating AKT/GSK3β/β-catenin axis in iCCA cells by using AB680, an inhibitor of CD73 enzymatic activity. AB680 treatment reduced p-AKT, p-GSK3β and β-catenin protein levels in a dose-dependent manner in CCLP1 cells. On the contrary, addition of exogenous adenosine activated the AKT/GSK3β/β-catenin signaling pathway in iCCA cells (Fig. 4F).

### CD73 inhibitor AB680 potentiates therapeutic efficacy of anti-PD-1 immunotherapy in murine iCCAs

**Fig. 5:**
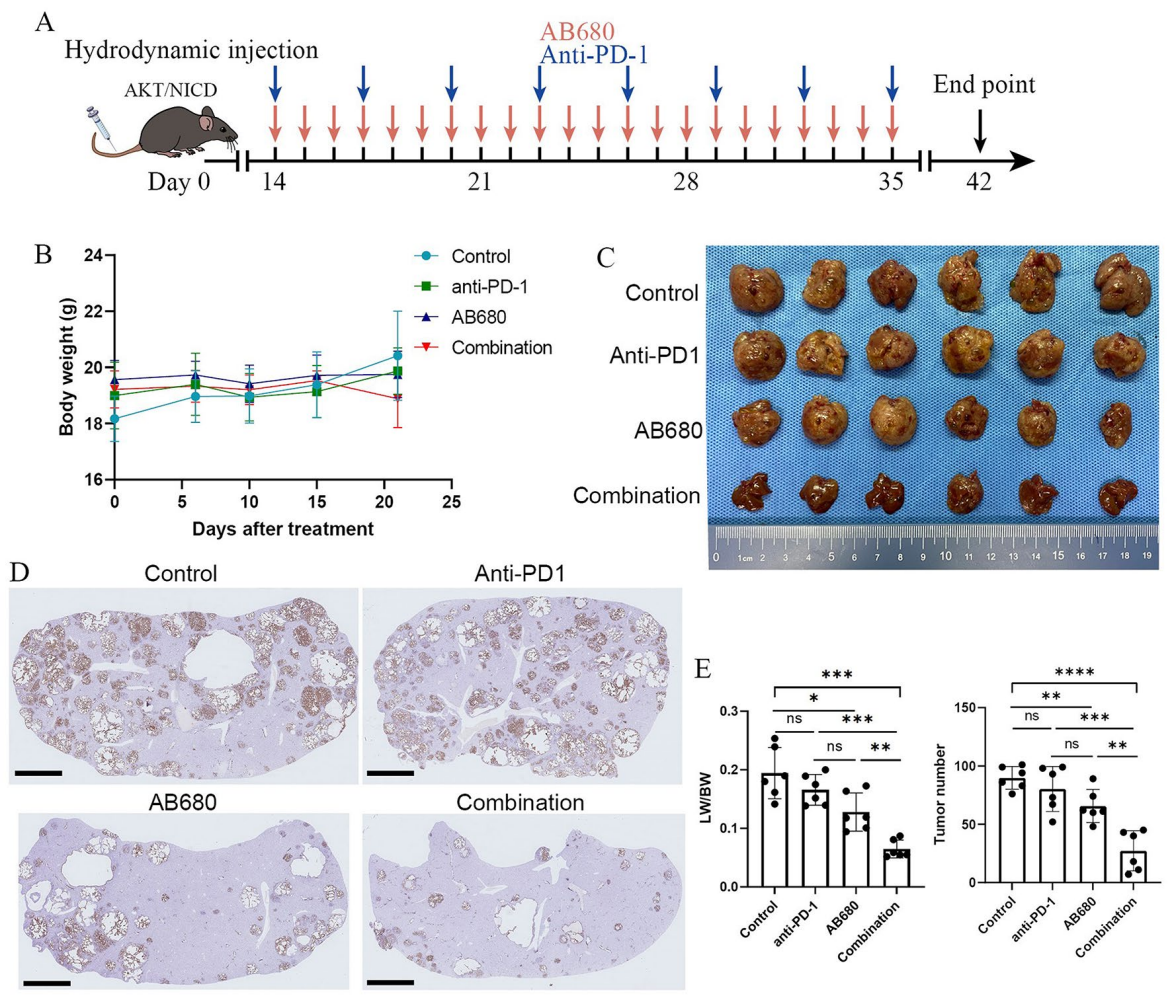
CD73 inhibitor AB680 potentiates therapeutic efficacy of anti-PD-1 immunotherapy in murine iCCAs (**A**) Schematic representation of the schedule for anti-PD-1, CD73 inhibitor AB680, or combination therapy in AKT/NICD-induced murine iCCA model. (**B**) Body weight of tumor-bearing mice treated with vehicle + 10 mg/kg IgG, 10 mg/kg anti-PD-1, 10 mg/kg AB680, or the combination therapy. (**C**) Liver images from spontaneous iCCA models that received the indicated treatments (6 mice per group) at the indicated endpoint. (**D**) Representative IHC staining images for CK19 of liver sections from AKT/NICD-driven murine iCCAs from indicted groups. (**E**) Statistical analysis of liver weight to body weight ratios (LW/BW) and tumor nodule numbers in AKT-NICD injected mice *P < 0.05, **P < 0.01, ***P < 0.001, ****P < 0.0001

Since high expression of CD73 induces immunosuppressive microenvironment in iCCA (SUN B Y et al. 2023) and anti-PD-1 therapy combined with other therapies such as Gem/Cis chemotherapy represents an effective treatment strategy for iCCA. We further assessed whether targeting CD73 could enhance the therapeutic activity of anti-PD-1 monoclonal antibodies (mAbs) using an AKT/NICD-induced spontaneous iCCA model. Two weeks after hydrodynamic tail vein injection, mice were divided into four groups: Control group (Vehicle + IgG), anti-PD-1 mAbs group (10 mg/kg), CD73 inhibitor AB680 group (10 mg/kg), and combination group, and then received indicated treatment following the schedule shown in Fig. 5A. Regular monitoring during drug administration showed no significant changes in body weight among the tested mice, indicating that the combination therapy had no obvious toxicity and side effects (Fig. 5B). In the AKT/NICD-induced spontaneous iCCA model, compared with monotherapy with anti-PD-1 antibody, AB680, or an IgG control, the combination therapy resulted in a significant reduction of tumor burden of primary iCCA revealed by the decreased liver weight versus body weight ratios (LW/BW) and number of tumor nodules (Fig. 5C-E). IHC analysis also confirmed the spontaneous induction of iCCA, as evidenced by biliary-specific cytokeratin (CK)-19 positive staining (Fig. 5D). Collectively, the combination therapy led to greater tumor regression than either AB680 or anti-PD-1 treatment alone.

### Combined treatment with CD73 inhibitor and PD-1 blockade activates anti-tumor immune response

**Fig. 6:**
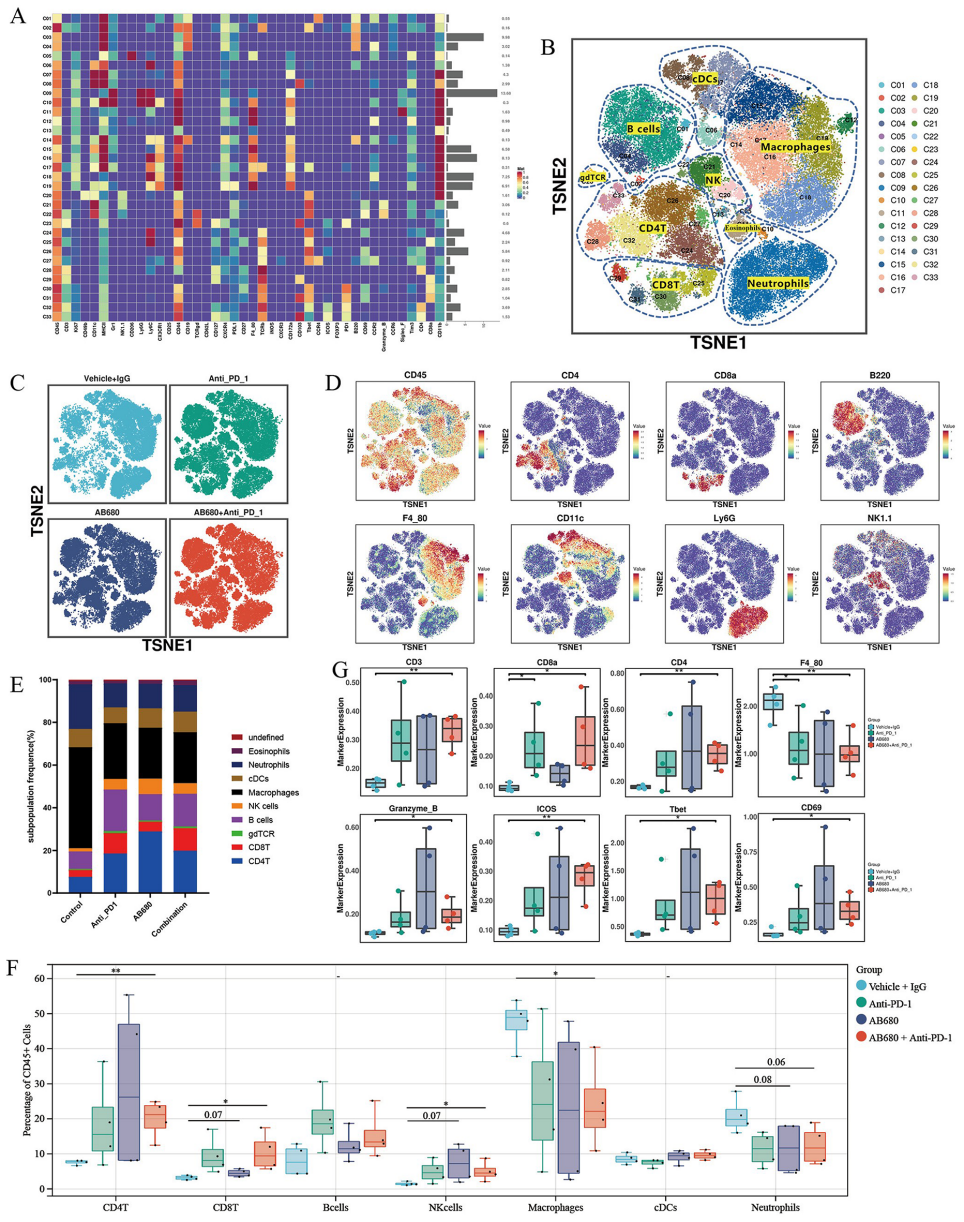
CD73 inhibition combined with anti-PD-1 therapy transforms the immune landscape of the TME (**A**) The heatmap showing the normalized expression of 42 surface and intracellular immune markers in all 33 immune-cell subclusters. (**B**) t-SNE plots of tumor-infiltrating B cells, macrophages, CD8^+^ T cells, CD4^+^ T cells, NK cells, neutrophils, and dendritic cells in the AKT/NICD-induced spontaneous murine iCCAs identified by CyTOF analysis. (**C**) t-SNE plots showing the major tumor-infiltrating immune cell populations from the four treatment groups (*n* = 4 per group). (**D**) t-SNE plots of tumor-infiltrating immune cells colored by the relative expression of corresponding lineage markers. (**E**) Stacked bar plots showing proportions of tumor-infiltrating immune cell populations identified by CyTOF analysis in each group. (**F**) Quantification of tumor-infiltrating B cells, macrophages, CD8^+^ T cells, CD4^+^ T cells, NK cells, neutrophils, and dendritic cells in AKT/NICD-induced iCCAs given the indicated treatment, assessed by CyTOF. (**G**) The expression level of indicated markers in tumor-infiltrating CD45^+^ immune cells among the four groups. *P < 0.05, **P < 0.01; TME, tumor microenvironment

Given that TIME dictates the treatment response to anti-PD-1-based immunotherapy, we conducted a high-dimensional characterization of the TIME landscape of the AKT/NICD-induced spontaneous murine iCCAs from the four treatment groups (*n* = 4) using time-of-flight mass cytometry (CyTOF). This metal-labeled antibody panel enabled us to assess the expression of 42 surface and intracellular immune markers on CD45^+^ immune cells from dissociated mouse iCCA tumors (Fig. 6A). Based on the expression of the corresponding lineage markers in each group, we identified the major tumor-infiltrating immune cell populations comprising B cells, macrophages, CD8^+^ T cells, CD4^+^ T cells, NK cells, neutrophils, and dendritic cells (Fig. 6B and D). The overall distribution of immune cells from each group was relatively uniform, with no obvious batch effects (Fig. 6C). Compared with the control group, the lymphoid cells of the other three groups were significantly increased, and the myeloid cells were significantly decreased, especially the macrophages (Fig. 6E). We then calculated the percentages of major tumor-infiltrating immune lineage in each individual sample, and found that compared with control group, the proportion of CD4^+^ T cells, CD8^+^ T cells and NK cells in tumors from AB680-treated group displayed an increasing trend, while the proportion of macrophages and neutrophils showed a decreasing trend (Fig. 6F). The major differences were observed between the combined treatment group and the control group. Combination therapy increased the proportion of CD8^+^ T cells, CD4^+^ T cells and NK cells, and decreased the proportion of macrophages and neutrophils (Fig. 6F). In parallel, T cell lineage markers CD3, CD8a, CD4 were increased, while macrophage markers F4/80 was decreased after combination treatment with anti-PD-1 and AB680. Besides, activation markers (GZMB, Tbet and CD69) and co-stimulatory factor ICOS were significantly upregulated on immune cells upon PD-1 blockade and CD73 inhibition (Fig. 6G).

**Fig. 7:**
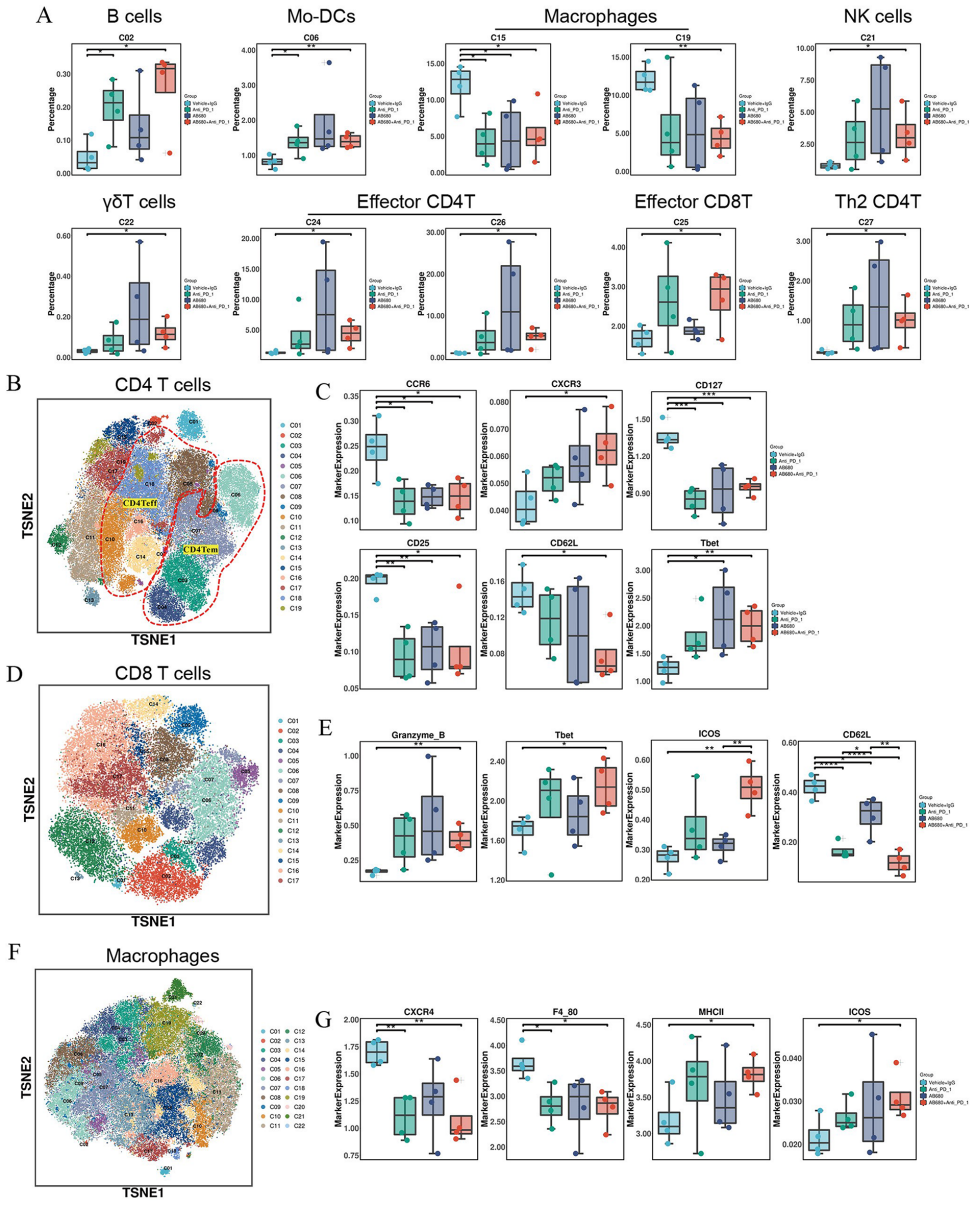
In-depth characterization of the immune-cell compartment by CyTOF analysis (**A**) Proportions of differential immune-cell subsets identified by CyTOF between the combination treatment group and control group. (**B**) Re-clustering analysis of CD4^+^ T cells according to the expression of selected 42 panel markers. (**C**) The expression level of indicated markers in tumor-infiltrating CD4^+^ T cells among the four groups. (**D**) Re-clustering analysis of CD8^+^ T cells and (**E**) expression level of indicated markers in tumor-infiltrating CD8^+^ T cells among the four groups. (**F**) Re-clustering analysis of macrophages and (**G**) expression level of indicated markers in tumor-infiltrating macrophages among the four treatment groups. *P < 0.05, **P < 0.01, ***P < 0.001, ****P < 0.0001

To further examine the specific immune cell subpopulations that might contribute to the anti-tumor effect of combination therapy, we compared the cellular proportions of the 33 clusters identified by CyTOF among the four treatment groups. In comparison to control group, the proportions of B cell subset (C02, MHCII^+^ CD44^+^ CD19^+^ Tbet^+^), dendritic cell (DC) subpopulation (C06, CD11c^+^ MHCII^+^ Ly6C^+^, Mo-DC), NK cell subpopulation (C21, NK1.1^+^ CD44^+^ Tbet^+^), γδT cells (C22, TCRgd^+^ Tbet^+^ GZMB^+^ CD69^+^), the effector CD4^+^ T cell subpopulations C24 (CD4^+^ CD44^+^ CD62L^−^ Tbet^+^) and C26 (CD4^+^ CD44^+^ CD62L^−^ Tbet^+^), effector CD8^+^ T cell cluster (C25, CD8a^+^ CD44^+^ CD62L^−^ Tbet^+^) and Th2 subpopulation (C27, CD4^+^ CD44^+^ CCR4^+^ Tbet^+^) were significantly enriched in the combination treatment group, whereas the proportions of the macrophage subpopulations C15 (F4_80^+^ SIRPα^+^ CX3CR1^+^) and C19 (F4_80^+^ Ly6C^+^ CXCR4^+^ PDL1^+^) were significantly decreased (Fig. 7A).

Re-clustering analysis of CD4^+^ T cells revealed that the expression of the TH17 marker CCR6, the Treg differentiation markers CD25 and CD127, and naïve T cell marker CD62L were significantly reduced in CD4^+^ T cells from the other three groups compared to the control group. Meanwhile, the expression of the functional activation marker Tbet was upregulated in CD4^+^ T cells from the AB680 monotherapy group, Tbet along with the Th1 marker CXCR3 was elevated in the combination therapy group (Fig. 7B-C). Similarly, re-clustering of CD8^+^ T cells showed that in comparison to control group, the expression of Granzyme B, Tbet, and the co-stimulatory factor ICOS was increased in CD8^+^ T cells from the combination therapy group, while the expression of the naïve T cell marker CD62L was consistently reduced in the other three groups (Fig. 7D-E). Analysis of macrophages indicated that the combination therapy decreased the expression of CXCR4 and F4_80, and increased the expression of MHCII and the co-stimulatory factor ICOS (Fig. 7F-G).

## Discussion

Our previous functional experiments revealed that CD73 promoted the malignant phenotypes of iCCA cells in vitro (SUN B Y et al. 2023). In this study, we further found that CD73 knockdown impeded growth of CCLP1 subcutaneous tumors in vivo, while CD73 overexpression accelerated tumor growth. To explore the underlying mechanism by which CD73 promotes iCCA progression, we performed RNA-seq analysis and western blot assays and found that CD73 overexpression in iCCA cells activated several downstream signaling pathways, such as the AKT/GSK3β/β-catenin signaling, which is known to promote proliferation, invasion, and migration of malignant cells and mediate tumor immune evasion. The impact of CD73 on oncogenic signaling in tumor cells mainly depends on the adenosine hydrolyzed from AMP by CD73. This is consistent with previous findings in HCC suggesting that CD73 promotes tumor progression and metastasis via activating PI3K/AKT signaling by the binding of adenosine to A2A receptor (MA X L, SHEN M N et al. 2019).

Several factors have been implicated in the upregulation of CD73 expression. Hypoxia-inducible factor-1 (HIF-1) can directly bind to the promoter region of NT5E thereby activating its expression (SYNNESTVEDT et al. 2002). Wnt/β-catenin signaling pathway could enhance expression of NT5E dependent on the presence of TCF-1 (SPYCHALA 2004). Soluble factors including type I IFNs (NIEMELA et al. 2004), TNFα (PAGNOTTA et al. 2013), IL-1b (SAVIC et al. 1990), IL-6 (ZENG et al. 2020), and TGF-β1 (REGATEIRO et al. 2011) are responsible for the upregulation of CD73 expression. CD73 is a direct target of miR-30a-5p (ZHU et al. 2017; ZHOU et al. 2019; XIE et al. 2017), miR-30d-5p (SONG and SONG 2018), and miR-193b, which are negative regulators of CD73 expression (MA X L, SHEN M N et al. 2019). Accumulating evidence shows that CD73 could also be a treatment-induced checkpoint. Radiotherapy (RT) increased surface expression of MHC class I molecules and immune checkpoint molecules PDL-1 and CD73 (WENNERBERG et al. 2020; SCHROTER et al. 2020). In a mouse model of T-cell immunotherapy, CD73 was induced in relapse melanomas, which acquired a mesenchymal-like phenotype. CD73 upregulation were also detected in melanoma patients progressing following adoptive T-cell transfer or ICBs, indicating an adaptive resistance mechanism to immunotherapy (REINHARDT et al. 2017). Chemotherapy with carboplatin, doxorubicin, gemcitabine, or paclitaxel induces enrichment of CD47^+^CD73^+^PDL1^+^ immune evasive breast cancer cells. These studies suggest that CD73 may be a therapy-induced checkpoint, and that CD73 blockade in combination with radiotherapy, chemotherapy, and immune checkpoint blockade might improve patient response to therapy (WENNERBERG et al. 2020). Our work substantiates CD73 as a promising target to combine with current chemotherapy or immunotherapies in iCCA. A combination of CD73 enzymatic inhibitor AB680 and GC chemotherapy achieved synergistic depression effects on iCCA growth than either single agent. Treatment with a selective CD73 inhibitor also significantly inhibited tumor growth and enhanced gemcitabine activity in pancreatic cancer (JACOBERGER-FOISSAC C, COUSINEAU et al. 2023). Pro-inflammatory cytokines have been reported to upregulate CD73 expression in the tumor microenvironment. Consistently, we found that ICBs therapy resulted in elevated expression of CD73 in malignant cells and identified TNF-α/NF-κB as positive transcriptional regulators of NT5E. However. IFN-γ stimulation showed no obvious effect on CD73 protein expression in iCCA cells.

Using AKT/NICD-induced spontaneous murine iCCA model, we found that anti-PD-1 therapy had limited suppressive effect on tumor initiation and progression, consistent with the clinical evidence that anti-PD-1 monotherapy is minimally effective in patients with iCCA. Anti-PD-1 therapy combined with other therapies like tyrosine kinase inhibitor (TKI) or Gem/Cis chemotherapy represents an effective treatment strategy for primary liver cancer including HCC and iCCA (MOK T S K, WU Y L, KUDABA et al. 2019; UENO et al. 2019; KUDO et al. 2018; ZHANG and HU 2021; YI et al. 2022). Since high expression of CD73 induces an immunosuppressive microenvironment in iCCA (SUN B Y et al. 2023) and the upregulated CD73 expression following ICBs therapy could impair the efficacy of ICBs, supporting the rationale for co-targeting CD73 and PD-1 to enhance the anti-tumor immune response, we queried whether this strategy applied to iCCA. We then assessed the efficacy of the combination therapy on immunocompetent spontaneous iCCA mouse models. While single AB680 agent treatment had modest anti-tumor effects, combining AB680 with anti-PD-1 mAbs revealed greater anti-tumor activity. Combination therapy with AB680 and anti-PD-1 therapy achieves a synergistic effect and transforms the immune landscape of tumor ecosystem in several type of cancers. AB680 improved the anticancer activity of immunosuppressive cells such as Treg and exhausted T cells in colorectal cancer (KIM et al. 2021). Small-molecule AB680-mediated inhibition of CD73 promoted CD8^+^ T-cell-mediated tumor regression in ductal pancreatic cancer (FARAONI E Y, SINGH 2023). CD73 inhibition reduced Treg accumulation and sensitized pancreatic cancer to PD-1 blockade (TANG et al. 2023). Our cytometry by time-of-flight analysis shows that CD73 inhibition combined with PD-1 blockade promoted the intra-tumoral infiltration of CD8^+^ T, CD4^+^ T cells and NK cells and simultaneously decreased the proportion of macrophages and neutrophils, suggesting that the combination therapy effectively activates anti-tumor immune response in iCCA.

## Conclusions

**Fig. 8:**
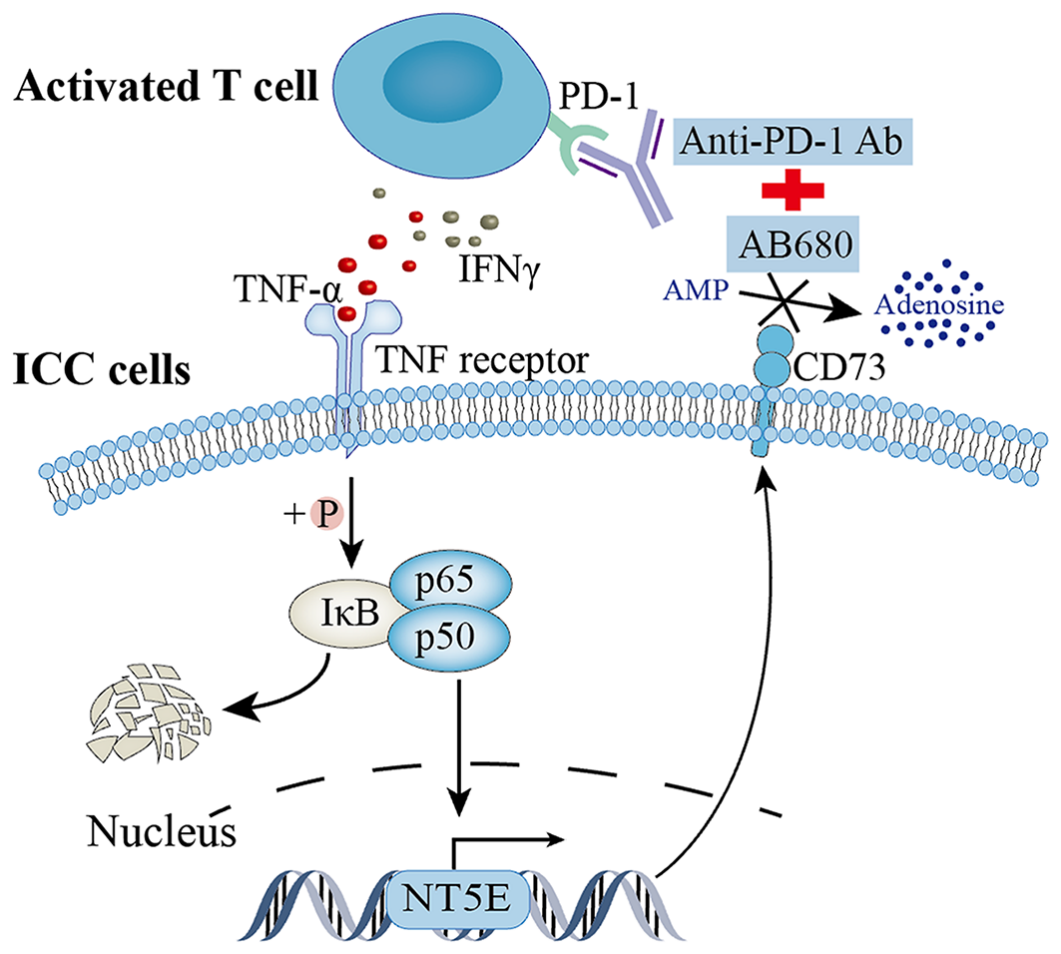
Schematic diagram depicting the rationale for combination therapy with CD73 inhibitor AB680 and PD-1 blockade

CD73 inhibitor AB680 exhibits potential as a promising anti-cancer treatment for iCCA, particularly through achieving a synergistic effect when combined with PD-1 blockade (Fig. 8). This combination therapy may contribute to the ongoing development of strategy for enhancing immunotherapy and GC chemotherapy efficacy in iCCA.

## Electronic supplementary material

Below is the link to the electronic supplementary material.

Supplementary Material 1

## Data Availability

All data generated during this study are available from the corresponding author upon reasonable request.

## Abbreviations

iCCA: Intrahepatic cholangiocarcinoma
ICBs: Immune checkpoint blockades
CyTOF: Time-of-flight mass cytometry
GC: Gemcitabine and cisplatin
NT5E: Ecto-5’-nucleotidase
PD-1: Programed cell death protein 1
DEGs: Differentially expressed genes
tSNE: T-distributed stochastic neighbor embedding
GSEA: Gene set enrichment analysis
OS: Overall survival
