# Supplementary material for: Targeting CD73 limits tumor progression and enhances anti-tumor activity of anti-PD-1 therapy in intrahepatic cholangiocarcinoma
Source: J Cancer Res Clin Oncol. 2024 Jul 13;150(7):348. doi: 10.1007/s00432-024-05869-1 (PMC11246275; doi:10.1007/s00432-024-05869-1)
Supplement: Supplementary file 1 — Supplementary Material 1 [file 432_2024_5869_MOESM1_ESM.docx]

| **Supplementary Table 1.** Antibodies used for CyTOF staining | | | | | |
| --- | --- | --- | --- | --- | --- |
| List | Label | marker | List | Label | marker |
| 1 | 89Y | CD45 | 22 | 159Tb | F4_80 |
| 2 | 115ln | CD3 | 23 | 160Gd | TCRb |
| 3 | 139La | Ki67 | 24 | 161Dy | iNOS |
| 4 | 141Pr | CD49b | 25 | 162Dy | CXCR3 |
| 5 | 142Nd | CD11c | 26 | 163Dy | CD172a |
| 6 | 143Nd | MHCII | 27 | 164Dy | CD103 |
| 7 | 144Nd | Gr1 | 28 | 165Ho | Tbet |
| 8 | 145Nd | NK1.1 | 29 | 166Er | CCR4 |
| 9 | 146Nd | CD206 | 30 | 167Er | ICOS |
| 10 | 147Sm | Ly6G | 31 | 168Er | FOXP3 |
| 11 | 148Nd | Ly6C | 32 | 169Tm | PD1 |
| 12 | 149Sm | CX3CR1 | 33 | 170Er | B220 |
| 13 | 150Nd | CD25 | 34 | 171Yb | CD69 |
| 14 | 151Eu | CD44 | 35 | 172Yb | CCR2 |
| 15 | 152Sm | CD19 | 36 | 173Yb | Granzyme_B |
| 16 | 153Eu | TCRgd | 37 | 174Yb | CCR6 |
| 17 | 154Sm | CD62L | 38 | 175Lu | Siglec_F |
| 18 | 155Gd | CD127 | 39 | 176Yb | Tim3 |
| 19 | 156Gd | CXCR4 | 40 | 197Au | CD4 |
| 20 | 157Gd | PDL1 | 41 | 198Pt | CD8a |
| 21 | 158Gd | CD27 | 42 | 209Bi | CD11b |
